# Improving the detection of *Helicobacter pylori* in biopsies of chronic gastritis: a comparative analysis of H&E, methylene blue, Warthin-Starry, immunohistochemistry, and quantum dots immunohistochemistry

**DOI:** 10.3389/fonc.2023.1229871

**Published:** 2023-07-17

**Authors:** Weisong Wan, Le Wang, Yufei Liu, Yuchang Hu

**Affiliations:** ^1^ The First College of Clinical Medical Science, China Three Gorges University, Yichang, China; ^2^ Institute of Pathology, China Three Gorges University, Yichang, China

**Keywords:** *Helicobacter pylori*, chronic gastritis, immunohistochemistry, methylene blue, Warthin-Starry, quantum dots immunohistochemistry

## Abstract

**Objectives:**

The objective of the study was to compare the consistency of various staining methods, including H&E, Methylene Blue, Warthin-Starry (W-S), Immunohistochemistry (IHC) and Quantum dots immunohistochemistry (QDs-IHC), in detecting *Helicobacter pylori* (HP) in cases of mild, moderate and severe chronic gastritis.

**Methods:**

Biopsy samples were obtained from 225 patients with chronic gastritis at the Department of Pathology, Yichang Central People’s Hospital between January 2019 and October 2019. The presence of HP was detected using H&E, Methylene Blue, W-S, IHC, and QDs-IHC.

**Results:**

The positive rates for HP detection using H&E, Methylene Blue, W-S, IHC, and QDs-IHC were 42.22%, 51.11%, 53.78%, 59.11%, and 58.67%, respectively. In cases of mild chronic gastritis, the consistency of test results between H&E, Methylene Blue, W-S, and QDs-IHC with IHC were Kappa=0.196, *P*=0.033, Kappa=0.706, P<0.001, Kappa=0.717, *P*<0.001, and Kappa=0.968, *P*<0.001, respectively. Similarly, in cases of moderate chronic gastritis, Kappa values between H&E, Methylene Blue, W-S, and QDs-IHC with IHC were 0.356, *P*<0.001, 0.655, *P*<0.001, 0.741, *P*<0.001, and 0.946, *P*<0.001, respectively. For cases of severe chronic gastritis, the Kappa values between the staining methods and IHC were 0.271, *P*=0.037, 0.421, *P*=0.002, 0.621, *P*<0.001, and 1, *P*< 0.001, respectively.

**Conclusion:**

The study showed that the positivity rate of IHC was significantly higher than that of H&E, Methylene Blue, and W-S in detecting HP infection in chronic gastritis cases. In terms of consistency with IHC, QDs-IHC was the most reliable staining method across all severity grades, while the agreement between H&E and IHC was poor, and that between Methylene Blue and W-S with IHC was average. Pathology departments may choose the most appropriate staining method based on their specific needs, considering the staining time, contrast, and cost of each method.

## Introduction

*Helicobacter pylori* (HP) is a gram-negative species of bacteria that reside between the mucosal and submucosal layers of the pyloric zone of the antrum. More than 50% of the global population is infected with HP (1). Recent studies have shown that HP infection is the leading cause of chronic gastritis and has been associated with peptic ulcers, gastric mucosa-associated lymphoid tissue (MALT) lymphoma, and gastric cancer (2–4). HP was designated as a class I carcinogen by the International Agency for Research on Cancer (IARC) in 1994, making it a clear carcinogenic agent. The Kyoto Global Consensus on HP gastritis and Maastricht Consensus on the Management of HP infection both define HP gastritis as a transmissible disease (5, 6). The most recent publication, “Screening and Eradication of *Helicobacter pylori* for Gastric Cancer Prevention: Taipei Global Consensus” promotes HP eradication strategies for gastric cancer prevention and features collaborative studies on population-wide screening and HP eradication initiatives (7). Therefore, the accurate diagnosis and treatment of HP infection is crucial in the diagnosis and treatment of the associated diseases, making precise detection of HP a significant topic of interest.

In clinical practice, the detection methods for HP can be mainly divided into invasive and non-invasive tests. Non-invasive tests, such as urea breath test, serum antibody detection, and stool antigen test, are relatively simple but may have a certain rate of misdiagnosis. Invasive tests include gastroscopy and histopathological examination using tissue samples obtained during gastroscopy to detect HP infection. Several methods for detecting HP from gastroscopic mucosal biopsy specimens are available in the pathology department, including H&E, special stains, immunohistochemistry (IHC), and Polymerase Chain Reaction (PCR) among others. PCR detection has high sensitivity, but due to its expensive cost and demanding experimental conditions, it is currently not widely available in some remote areas with poorer conditions. Through continuous practice, the IHC method has become the main method used by the author’s department to assist in detecting the infection status of HP due to its excellent performance. Recently, QDs-IHC has also been applied to the detection of HP in gastric mucosal biopsies. However, studies assessing the utility of these various tests in patients with various degrees of inflammation are scarce. The main objective of this manuscript is to discuss various common adjunct diagnostic methods employed in pathology departments for the detection of HP. The study aimed to assess the presence of HP infection in gastroscopic mucosal biopsy samples utilizing H&E, Methylene Blue, W-S, IHC, and QDs-IHC techniques. IHC was used as the gold standard for the first time to analyze the consistency of four other methods with IHC in detecting HP results in different types of chronic gastritis, in order to evaluate the superiority and inferiority of these detection methods. This research will provide a certain basis for selecting a precise and practical HP detection method for clinical diagnosis.

## Subjects and methods

### Study material

Chronic gastritis can mainly be classified into the following types according to etiology and pathological characteristics: 1) Hp-related chronic gastritis, which is a common type associated with Hp infection; 2) Autoimmune chronic gastritis, which is rare and related to immune abnormalities; 3) Drug-related chronic gastritis, such as non-steroidal anti-inflammatory drugs and indomethacin-induced chronic gastritis; 4) Radiation-induced chronic gastritis, which occurs after radiation therapy; 5) Idiopathic chronic gastritis, which is a minority of chronic gastritis with unknown etiology (8). This manuscript screened out cases diagnosed with chronic gastritis by searching the department’s information system, while excluding cases mentioned in items 2 to 5 above. From January 2019 to October 2019, a total of 225 (determined through a power analysis, taking into account an estimated effect size, desired statistical power, and significance level) gastric mucosal biopsies were collected from patients diagnosed with chronic gastritis at the Department of Pathology, Yichang Central People’s Hospital. All screened cases were classified into mild, moderate, and severe chronic gastritis according to the literature (9). All specimens were fixed in 10% neutral buffered formalin, routinely dehydrated, embedded in paraffin, and serially sectioned at 4 μm thickness. Adherent slides were laid in a consistent orientation and stained with H&E, Methylene Blue, W-S, IHC, and QDs-IHC. This study was approved by the ethics committee of the People’s Hospital of Yichang city. All cases were obtained with the written consent of the patients themselves or their families members.

### H&E staining

H&E staining was performed according to the routine laboratory procedures using an H&E automated stainer (Thermo, USA). H&E were prediluted, reagents purchased from Zhuhai Beso Biotechnology Co., LTD.

### Methylene blue staining

Methylene Blue staining was performed according to the manufacturer’s instructions (Beso, Zhuhai, China). Paraffin sections were routinely deparaffinized to water. Staining was performed using distilled water cleaning followed by dropwise addition of Methylene Blue staining solution (prediluted, Beso, Zhuhai, China) for 10 minutes. Excess dye solution was removed by washing with distilled water. Specimens were air dried using an electric blower before being transparent with xylene and sealed with neutral gum. Positive and negative controls were set up simultaneously to ensure the accuracy and objectivity of the study.

### W-S staining

W-S staining was performed according to the manufacturer’s instructions (Beso, Zhuhai, China). Paraffin sections were routinely deparaffinized to water, washed with deionized water, and immersed in staining solution (prediluted, Beso, Zhuhai, China). The sections were then placed in a water bath box at 56°C to react for 30-60 minutes. After being removed without water washing, the sections were placed on a staining rack and the pre-prepared developing solution was added for 10-20 seconds. The sections were then washed with preheated deionized water at 56°C when they appeared gold-yellow or yellow-brown. After dehydration with absolute ethanol, they were transparent with xylene and sealed with neutral gum. Positive and negative controls were set up simultaneously to ensure the accuracy and objectivity of the study.

### IHC staining

IHC was performed using the EnVision two-step method. Deparaffinized sections were stripped and repaired in a high-pressure pot with repair solution containing EDTA (pH 9.0) for three minutes. A 3% hydrogen peroxide solution was soaked for 15 minutes to eliminate peroxidase activity. Monoclonal mouse anti-HP antibody (prediluted, Maixin, China) was added dropwise and incubated for 60 minutes at room temperature. The secondary antibody was horseradish peroxidase labeled anti-mouse/rabbit IgG (prediluted, Dako, Denmark). After incubation for 30 minutes at room temperature, diaminobenzidine (DAB) chromogen solution was added dropwise for seven minutes at room temperature, and finally, the nuclei were counterstained with hematoxylin. Positive and negative controls were set up simultaneously to ensure the accuracy and objectivity of the study.

### QDs-IHC staining

Deparaffinized sections were stripped and repaired in a high-pressure pot with repair solution containing EDTA (pH 9.0) for three minutes. After incubation in 2% BSA buffer (Sigma, USA) at 37°C for 30 minutes, monoclonal mouse anti-HP antibody (prediluted, Maixin, China) was added dropwise and incubated at 37°C for 60 minutes. The sections were then incubated in 2% BSA buffer at 37°C for another 10 minutes. Quantum dot-labeled goat anti-mouse IgG-525 (prediluted, Jiayuan, China) was subsequently added for incubation at 37°C for 50 minutes. The sections were blocked with 90% glycerol (Sigma, USA). Positive and negative control tissues were added to ensure the accuracy and objectivity of the study.

### Interpretation of results

H&E, Methylene Blue, W-S, and IHC staining results were observed under a light microscope, while QDs-IHC results were observed under a fluorescence microscope. All staining results were interpreted by two independent observers with senior titles in a double-blind manner. If the interpretations were inconsistent, a third independent observer was consulted. The positive signals of HP were classified into four grades (0, 1+, 2+, and 3+) according to the pathological diagnostic criteria of chronic gastritis of China and the new Sydney system by visual analogue scoring (9–11).

### Statistical analysis

Paired chi-square test and Kappa test were performed by SPSS 20.0 to analyze the data. Kappa < 0.40 represented poor consistency, 0.40 ≤ Kappa < 0.75 represented consistency in general, and Kappa ≥ 0.75 represented good consistency (12). The level of statistical significance was set at *P* < 0.05. Sensitivity and specificity were calculated based on the following formulae: Sensitivity= (True positive/(True positive + False negative)) * 100%; Specificity= (True negative/(True negative + False positive)) * 100% (13).

## Results

### Clinical features

A total of 225 cases of chronic inflammation gastric mucosal biopsy cases were screened through database retrieval and review slices. Among them, 123 cases (54.67%) were males aged 20-75 with an average age of (51.6 ± 11.2) years old, and 102 cases (45.33%) were females aged 27-79 with an average age of (52.8 ± 10.4) years old. Of the total cases, 83 (36.89%) demonstrated mild chronic gastritis, 94 (41.78%) displayed moderate chronic gastritis, and 48 (21.33%) showed severe chronic gastritis.

### Morphology and localization of HP with different detection methods

**Figure 1:**
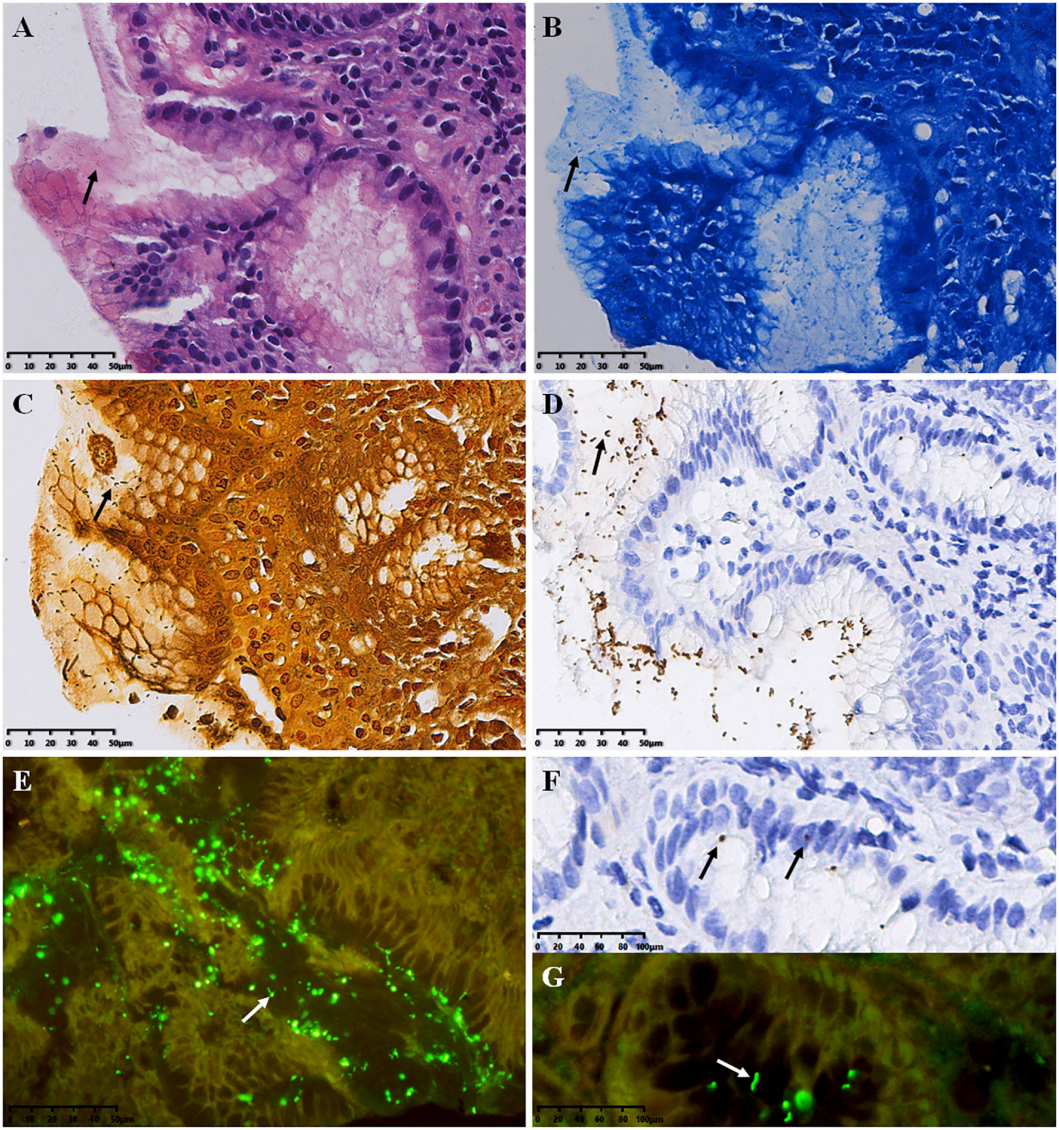
Results of HP detection (arrows refer to HP bacteria) **(A)** H&E stain, **(B)** Methylene Blue stain, **(C)** W-S stain, **(D, F)** IHC stain, envision two-step staining; **(E, G)** QDs-IHC staining, indirect staining. All images are from the same severe chronic gastritis case. Original magnifications:×300 **(A–E)**, ×600 **(F, G)**.

HP positive signals were mainly distributed in the gastric pit cavity and mucus on the mucosal surface, which appeared spiral, curved or short rod-shaped. In H&E staining, HP was observed as rod-shaped, S-shaped or small dots (**Figure 1A**). In Methylene Blue staining, HP exhibited a blue S-shaped, short rod-shaped, or small dot-shaped appearance, with the background tissue staining blue (**Figure 1B**). In W-S staining, HP appeared as brown to black, while the rest of the background tissue stained yellow to brown (**Figure 1C**). In IHC staining, HP appeared yellow or brown spiral, S-shaped, or short rod-shaped (**Figure 1D**); occasionally, coccoid forms of HP were observed in the epithelial cell layer and the lamina propria of the gastric mucosa (**Figure 1F**). QDs-IHC staining showed high-brightness green fluorescence under a fluorescence microscope, appearing as a short rod shape, spiral-shaped, or dot-shaped. HP predominantly localized in the gastric mucosal tissue’s epithelial surface and gastric pits, with a small amount located in the glandular cavity close to the mucosa (**Figures 1E, G**).

### Statistics of HP detection results among different detection methods in different degrees of inflammation

**Figure 2:**
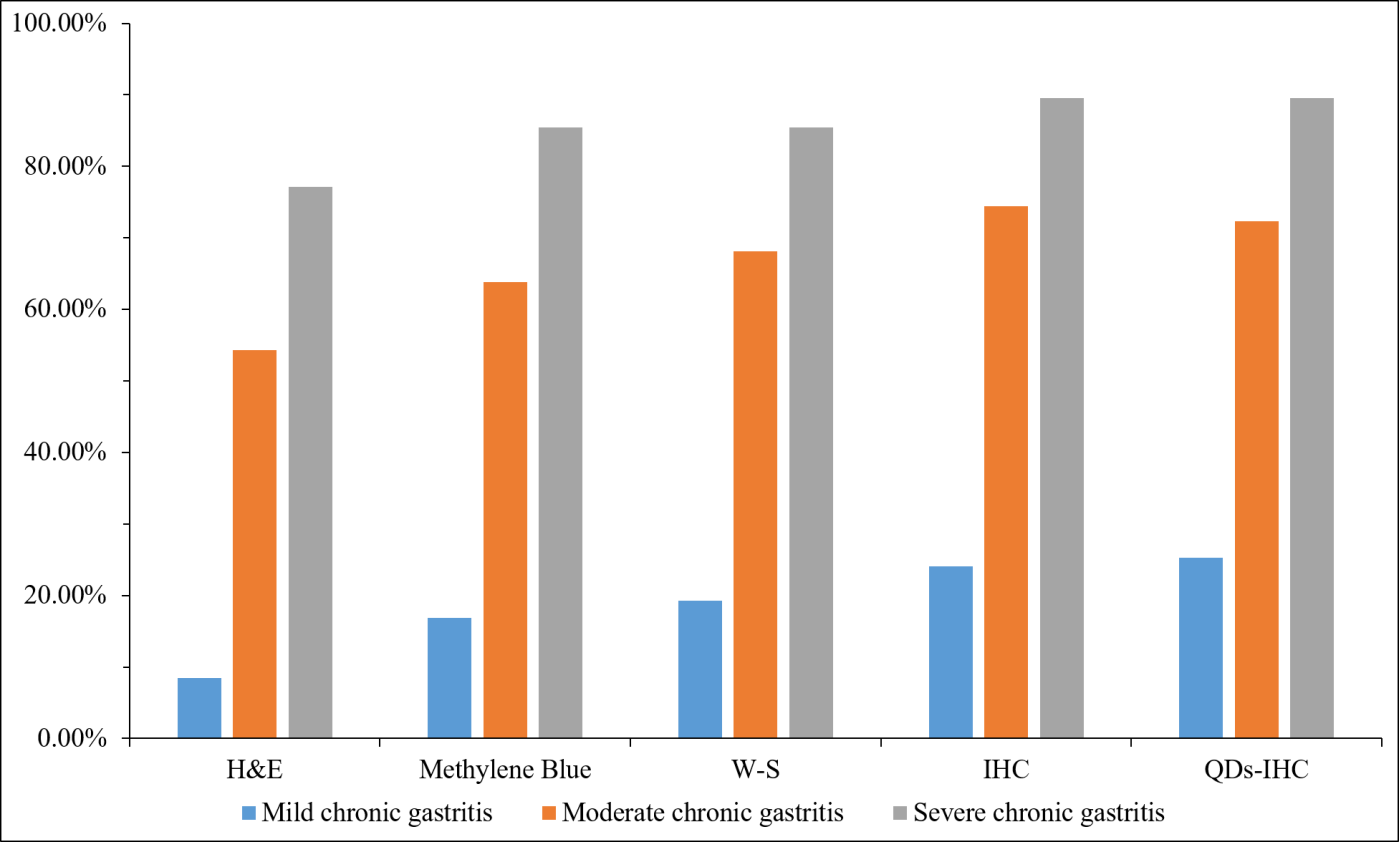
Statistics of different detection methods in patients with mild, moderate and severe chronic gastritis.

As show in **Figure 2**. The HP positivity of H&E staining was 95 (42.22%) in 225 chronic gastritis cases, 7 (8.43%), 51 (54.26%) and 37 (77.08%) in mild, moderate and severe chronic gastritis, respectively. That of Methylene Blue staining was 115 (51.11%) in total, 14 (16.87%), 60 (63.83%) and 41 (85.42%) in mild, moderate and severe chronic gastritis, respectively. That of W-S staining was 121 (53.78%) in total, 16 (19.28%), 64 (68.09%) and 40 (85.42%) in mild, moderate and severe chronic gastritis, respectively. Overall HP positivity by IHC staining was 133 (59.11%) and 20 (24.10%), 70 (74.47%) and 43 (89.58%) in mild, moderate and severe inflammation cases, respectively. Overall HP positivity by QDs-IHC staining was 132 (58.67%) and 21 (25.30%), 68 (72.34%) and 43 (89.58%) in mild, moderate and severe inflammation cases, respectively. These data showed that the positivity rate of IHC was significantly higher than that of H&E, Methylene Blue, and W-S in detecting HP infection in chronic gastritis cases.

### Consistency analysis of detection results among different detection methods

**Table 1** shows the consistency of detection results among H&E, Methylene Blue, W-S, and QDs-IHC with IHC in all cases of chronic gastritis. **Table 2** displays the consistency of detection results among H&E, Methylene Blue, W-S, and QDs-IHC with IHC in mild, moderate, and severe chronic gastritis. These results suggest that in terms of consistency with IHC, QDs-IHC was the most reliable staining method across all severity grades, while the agreement between H&E and IHC was poor, and that between Methylene Blue and W-S with IHC was average.

**Table 1:** Consistency analysis of five different HP detection methods in chronic gastritis.

Detection methods		IHC		*Kappa*	*P*
		Positive	Negative		
H&E	Positive	85	10	0.499	<0.001
	Negative	48	82		
Methylene Blue	Positive	110	5	0.750	<0.001
	Negative	23	87		
W-S	Positive	116	5	0.802	<0.001
	Negative	17	87		
QDs-IHC	Positive	131	1	0.972	<0.001
	Negative	2	91		

**Table 2:** Consistency analysis of five different HP detection methods in different degrees of gastritis.

Degree of inflammation	Detection methods		IHC		*Kappa*	*P*
			Positive	Negative		
Mild chronic gastritis	H&E	Positive	4	3	0.196	0.033
		Negative	16	60		
	Methylene Blue	Positive	13	1	0.706	<0.001
		Negative	7	62		
	W-S	Positive	14	2	0.717	<0.001
		Negative	6	61		
	QDs-IHC	Positive	20	1	0.968	<0.001
		Negative	0	62		
Moderate chronic gastritis	H&E	Positive	46	5	0.356	<0.001
		Negative	24	19		
	Methylene Blue	Positive	58	2	0.655	<0.001
		Negative	12	22		
	W-S	Positive	62	2	0.741	<0.001
		Negative	8	22		
	QDs-IHC	Positive	68	0	0.946	<0.001
		Negative	2	24		
Severe chronic gastritis	H&E	Positive	35	2	0.271	0.037
		Negative	8	3		
	Methylene Blue	Positive	39	2	0.431	0.002
		Negative	4	3		
	W-S	Positive	40	1	0.621	<0.001
		Negative	3	4		
	QDs-IHC	Positive	43	0	1	<0.001
		Negative	0	5		

## Discussion

The incidence of HP infection is closely related to the level of socioeconomic development and sanitation. Research indicates that the natural population of China has a 54.76% infection rate (14). HP-related gastritis is one of the most common infectious diseases and the most important cause of gastric cancer in China. Gastric cancer ranks fifth among the top ten cancers worldwide with a death toll of 770,000 in 2020 (15). Early detection and effective treatment of HP-associated gastritis is crucial in preventing gastric cancer (2, 4, 16, 17). Pathology departments use various methods to diagnose HP, including morphology-based H&E, Methylene Blue, and W-S stains, immunology-based IHC and QDs-IHC, and gene-based PCR tests. PCR require expensive experimental conditions that limit their usage in remote areas and third-world countries. This research aimed to compare the consistency of H&E, Methylene Blue, W-S, IHC, and QDs-IHC in detecting HP in biopsy specimens of chronic gastritis, providing a basis for selecting the appropriate detection method for clinical diagnosis of HP-related gastritis.

H&E staining requires high-power microscopy to accurately observe HP morphology, which may be indistinguishable from impurities and contamination during staining. The positive rate of HP varies greatly in different laboratories. Only pathologists who have undergone rigorous training and follow a consistently optimized preparation process can guarantee accurate detection of HP. The results of this study showed that HP in H&E was pale and had poor contrast. When there was only a small amount of HP infection, the diagnosis was often missed due to the difficulty of observation. The false positive and false negative results of H&E staining were 10 (7.5%) and 48 (52.17%), respectively (**Table 1**). Therefore, the sensitivity and specificity of H&E staining were only 63.91% (85/(85 + 48)) and 89.13% (82/(82 + 10)). Some studies show that H&E has poor specificity with high false positives and false negatives (18), which is consistent with our results.

Methylene Blue staining has several advantages, including fewer staining steps, less time-consuming procedures, ease of operation, and low cost. However, it is important to be cautious when examining the staining results under a high magnification microscope, as the HP and background cells both present as blue. To address this, some studies have modified Methylene Blue staining, making it easier to observe the surface near the mucosal cells under the mucus layer in order to identify any changes in HP morphology. Despite these modifications, potential diagnosis oversights remain a concern as it is easy to miss changes in HP morphology. For example, El-zimaity et al. evaluated gastric biopsies from 200 patients treated for HP infection and found that Methylene Blue staining had up to 11% false negatives (19). Similarly, in this study, we found that Methylene Blue staining had a 25% (23/92) false negative rate, particularly in weakly positive (+) cases like those observed via H&E staining. The sensitivity and specificity of methylene blue staining were 82.71% (110/(110 + 23)) and 94.57% (87/(87 + 5)) (**Table 1**).

W-S staining reveals HP bacteria as brown to black, providing high contrast against the yellowish-brown background. However, this method requires complex preparation of the dye solution, specific temperature control, and prolonged dyeing time, with stringent external environmental demands. The presence of black silver particles can lead to false positive results if not discriminated carefully from HP bacteria. In our study, the false positive rate of W-S staining was 3.76% (5/133), comparable to the 6% rate reported by Rotimi et al. (20), but higher than IHC staining. The sensitivity and specificity of W-S staining were 82.71% (116/(116 + 17)) and 94.57% (87/(87 + 5)) (**Table 1**).

The IHC staining of HP results in a positive brown signal, which stands out against the light blue nuclear background observed after restaining with Hematoxylin. The positive signal can be easily observed in the gastric pits and glandular lumen of the gastric mucosa, even under low-power microscope. However, the increased use of antibiotics and proton pump inhibitors has brought about significant changes in the characteristics of HP infection (21, 22). The number of HP that has been exposed to these adverse environmental conditions has reduced, making it more challenging to locate them. Those that remain may be found in deep recesses or glands, or even migrate to the proximal region of the stomach, making it difficult to recognize them through morphological observation using H&E, Methylene Blue, and W-S staining. Instead, the use of the IHC method, based on antibody-antigen binding, allows for the easy identification of spherical HP and even small amounts of HP in the gastric epithelium and stroma. In addition to aiding in the identification of a small amount of HP on the surface of caveolae and mucosa (**Figure 1D**), the IHC method can also be used to observe HP bacteria and deformed components of HP within stromal and epithelial cells (**Figure 1F**).

QDs are a type of nano-fluorescent dye that has been developed in recent years. They have significant advantages over traditional fluorescent dyes, such as good light stability and high fluorescence intensity. Due to their excellent physical and chemical properties, QDs are increasingly used in bioimaging (23), molecular and cell labeling (24, 25) and *in vivo* labeling (26). This study shows that QDs-IHC staining offers several benefits, including high-brightness green fluorescence, clear contrast with the background, excellent sensitivity, and specificity, similar to the IHC method. With QDs-IHC, even a small number of bacteria can be easily detected under a fluorescence microscope. Although, it requires the use of antibodies labeled with quantum dots and a fluorescence microscope for observation, which may not be suitable for basic hospitals lacking adequate equipment. Moreover, patients will incur higher costs and greater burden.

The results of the concordance analysis reveal that the agreement between H&E and IHC staining was inadequate for mild, moderate, or severe chronic gastritis. The agreement between Methylene Blue and W-S staining and IHC staining was moderate, while the agreement between QDs-IHC and IHC staining was satisfactory. Therefore, relying solely on H&E staining to diagnose the presence of HP infection in gastric biopsy cases, regardless of the severity of gastritis, is highly unreliable. Methylene blue staining and W-S staining are also insufficient in detecting HP infection adequately. Only the use of IHC or QD-IHC methods can accurately diagnose HP infection to the greatest extent possible.

This study, however, does have certain limitations. For instance, it only entails a comparative analysis of various detection methods conducted within a single laboratory of the author’s department. Thus, a multi-center comparative analysis with a larger sample size would yield more compelling results. Additionally, clinicians and pathologists must acknowledge that even with the highest positive rate for IHC testing, a negative result does not necessarily mean the absence of HP infection. This is related to factors such as whether the sample is fully adequate and whether the section is sufficient. In addition, a larger sample size, stricter selection criteria for cases, and diverse independent observers’ interpretations would enhance the persuasiveness of the results. We will consider conducting multi-center comparative analysis in future studies, involving stricter selection criteria for cases and further increasing the sample size. Additionally, we will select diverse observers, including those with different professional levels, to interpret the results.

In summary, each detection method has its own set of advantages and disadvantages. Pathologists can assess the staining time, staining contrast, and cost of different staining methods and select a detection method that is best suited to their requirements. Histochemical staining may present challenges in detecting rare spherical HP, particularly if they are located deep within the gland or inside the cell. When diagnostic features are unclear in H&E staining, such as in cases where the number of bacteria is small, or when tissue is rare or exhibits morphological changes, confirmatory IHC staining analysis can be performed to improve diagnostic accuracy. Additionally, if possible, QDs-IHC staining analysis can also be considered.

## Data availability statement

The original contributions presented in the study are included in the article/supplementary material. Further inquiries can be directed to the corresponding author.

## Ethics statement

The studies involving human participants were reviewed and approved by the ethics committee of the People’s Hospital of Yichang city. The patients/participants provided their written informed consent to participate in this study.

## Author contributions 

WW performed experiments, analyzed of the results and wrote the manuscript. YL performed the data analysis and provided writing assistance. YH contributed to the design of the study and provided writing assistance. LW helped performed the experiments and the data analysis. All authors read and approved the final manuscript. All authors contributed to the article and approved the submitted version.
